# Predictors of in-hospital mortality among under-five children with severe acute malnutrition in South-Western Uganda

**DOI:** 10.1371/journal.pone.0234343

**Published:** 2020-06-26

**Authors:** Timothy Nduhukire, Daniel Atwine, Luwaga Rachel, Joseph E. Byonanebye

**Affiliations:** 1 Department of Pediatrics and Child Health, Kabale University, Kabale, Uganda; 2 SOAR Research Foundation, Mbarara, Uganda; 3 Department of Nursing, Bishop Stuart University, Mbarara, Uganda; 4 Department of Biomedical Sciences, Marquette University, Milwaukee, Winconsin, United States of America; Azienda Ospedaliero Universitaria Careggi, ITALY

## Abstract

**Background:**

Severe acute malnutrition (SAM) affects about 13 million under-five children (U5), with an estimated one million dying every year. In this study we aimed at determining the in hospital mortality and its associated factors among U5s admitted with SAM.

**Methods:**

This was a prospective cohort study of children 6 months to 5 years with SAM admitted at Mbarara Regional Referral Hospital (MRRH) between June and August 2015. Care-takers were interviewed to collect socio-demographic and clinical information. Children under-went physical examination and had blood drawn for HIV, serum glucose, malaria, full blood count, culture and serum electrolytes investigation. Children were managed according to WHO treatment guidelines for SAM. All participants were followed up for a maximum period of 30 days. The proportion of U5 deaths within the first 48 hours and during the entire admission period was calculated. Using Poisson regression analysis, predictors of in-hospital mortality were analyzed with STATA/IC 11.0.

**Results:**

We enrolled 122 children, median age of 15 months [IQR:11–24], 58.2% males, 90% immunized, 81% ill for more than 2 weeks before admission, 71% from lower health facilities and majority with unknown HIV status(76%). Overall, 13 (10.7%) children died in hospital. Seven (5.7%) died within the first 48 hours. Intravenous (IV) fluid administration significantly predicted in-hospital mortality (adjusted IRR: 7.2, 95%CI: 2.14–24.08, *p = 0*.*001*).

**Conclusion:**

The in-hospital mortality in U5s with SAM was lower than that previously reported in central Uganda. Intravenous fluid administration significantly predicted overall in-hospital mortality. While Administration of intravenous fluids is still the main stay of managing severely malnourished children with shock, more research needs to be conducted in order to review the parameters presently used to assess children for shock with a view of diagnosing and managing shock in these children when it is still early. Adequate guidance on use of IV fluids in management of severely malnourished children should be prioritized during continuous medical education for healthcare workers and in the treatment guidelines.

## Background

Childhood malnutrition, is the imbalance between nutrient requirement and intake, resulting in cumulative deficits of energy, protein, or micronutrients that may negatively affect growth, development, and other relevant outcomes [1]. Child undernutrition is a major public health problem, especially in many low-income and middle- income countries [2]. Poor nutrition is associated with suboptimal brain development, which negatively affects cognitive development, educational performance and economic productivity in adulthood [3]. The African region and South-East Asia have reported the highest prevalence of undernutrition, with the former accounting for about 39.4% of the stunted, 24.9% of the underweight and 10.3% of the wasted children under-5 years of age [4]. Severe acute malnutrition (SAM) as defined by World Health Organization-United Nations Children’s Fund includes severe wasting and nutritional edema. Severe wasting is defined as weight-for-height below −3 SD or MUAC <115mm [5]. About 25 to 35 million under-five children are living with SAM worldwide [6]. Thirteen Million of these children live in Sub Saharan Africa [6]. Of these 13 Million, I million will die every year [6]. The worst affected parts of Africa are west, central and eastern Africa [6]. In East Africa, Uganda in particular has been reported to have the highest child mortality rates in the world [7]. 40% of these deaths have been attributed to malnutrition as the underlying cause [7].

The World Health Organisation (WHO) has developed management guidelines that if strictly followed, should be able to reduce the mortality to less than 10% [8]. Mortality from SAM has however persistently remained between 10 and 40% in many hospitals in Sub Saharan Africa, despite use of these guidelines [9–13]. This high mortality has been commonly attributed to HIV infection, lack of maternal participation in the feeding program, inadequate care and prescription errors, and over prescription of intravenous therapies and blood transfusion [9, 11, 12, 14–16]. Another study attributed the high mortality in children admitted with SAM to the WHO identified danger signs; lethargy, hypoglycemia and hypothermia as well as bradycardia, capillary refill greater than 2 seconds, weak pulse volume and impaired level of consciousness [10]. Overall, few studies have been done in Uganda to determine the risk factors for mortality in these children. No such study has been done in Western Uganda, yet this region has the highest prevalence of Acute Malnutrition in Uganda [17].

In this study, we aimed at determining the in-hospital mortality rate and its predictors among U5s admitted with SAM on the Pediatric ward at Mbarara Regional Referral Hospital (MRRH), so as to define the most at-risk group of children that need to be triaged for prompt emergency monitoring and treatment during their admission in hospital.

## Methods

### Study setting

The study was conducted at Mbarara Regional Referral Hospital on the Pediatrics ward. The hospital, which is 269 km from Kampala serves as a regional referral hospital for south western Uganda. Its catchment area includes the districts; Buhweju, Bushenyi, Ibanda, Isingiro, Kiruhura, Mitooma, Ntungamo, Rubirizi, Sheema and Mbarara which are all located in south western Uganda whose population is mainly rural with many socio economic challenges.

### Study design

We conducted a prospective cohort study between June and August 2015 and enrolled 122 children aged 6 months to 5 years with a diagnosis of SAM at admission to the pediatric ward of MRRH.

### Participants

A child was considered eligible if he or she had features of SAM at admission, that is, a weight for length z score < -3SD, MUAC of <11.5 cm or bilateral edema, [18] and if their caretaker provided a signed written informed consent. A child was excluded from enrolment to the study if he or she had Nephrotic syndrome, liver disease or heart disease based on medical history, physical exam or investigations.

### Study procedures

At enrolment in the study, a pretested structured questionnaire was administered in the local language to caretakers and used to capture information on: socio-demographic characteristics of both caretaker and U5s specifically age, sex, district of residence, marital status and occupation; U5s’ medical history and pre-admision care; U5s clinical presentation; and care received at and during admission in hospital.

Children underwent a general examination with special emphasis on axillary temperature, presence of visible wasting, bilateral edema, eye signs of vitamin A deficiency, pallor of mucous membranes and presence of severe dehydration. Systemic examination was done with special emphasis on the cardiovascular system where pulse rate, capillary refill time, temperature gradient in the limbs was assessed, and the central nervous system where the level of consciousness was noted. Pulse rate and oxygen saturation was done using an ANP100 pulse oximeter (made by Ana Wiz Ltd, Units 5–6, UK, 2012). The weight and length/height was determined, weight for height z scores plotted and presence of bilateral edema ascertained to make a diagnosis of SAM Length was measured with the child lying supine on a measuring board and recorded to nearest 0.1 cm. Weight was measured by having child undressed and placed on a digital infant scale (Seca 384) or adult scale (Seca 762) if the child was able to stand. Weight was recorded to nearest 0.1 kg. Weight for height z scores were determined using WHO 2006 growth charts. At enrolment, a total of 4 ml of blood were drawn under sterile conditions during insertion of IV cannula for measurement of full blood count, blood glucose, blood culture, serum electrolytes, blood slide for malaria and HIV test. Specifically, prior to drawing of blood, the area was swabbed with cotton dipped in ethyl alcohol 70% to prevent contamination. Incase of HIV test, pre- and post-test care-taker counseling was done. A first Determine® HIV rapid test was performed and confirmed with a 2nd rapid test StatPak®. In case of discordance, a Unigold® test was done. Children under 18 months with positive rapid tests were referred for DNA PCR test in the HIV clinic at MRRH.

Children with SAM were managed by pediatricians, doctors, nurses and nutritionists involved in child health care at the pediatric unit of MRRH following routine practices based on the updated WHO guidelines (Organization, 2013). The research team did not in any way interfere in the management of these children while in hospital. All children were routinely treated with IV Ampicillin and IV Gentamycin for the first 5 days. They were also routinely started on a feeding program with F75 formula feeds. Caretakers took responsibility for the feeding of the children under the supervision of the hospital nutritionist. Children with diarrhea were routinely given Zinc tablets in addition to the routine deworming tablets that were given to all the enrolled patients. Those with some dehydration were given Resomal solution while those with severe dehydration or septic shock were given IV half strength Darrows/5% Dextrose solution.

All participants were followed-up by the research team for a maximum period of 30 days. During this period, the research team kept in contact with the healthcare workers to ascertain clinical progress of study participants. Information with regard to any death or disappearance of child due to self-discharge by caretakers, was relayed by the managing healthcare workers to the principal investigator who would then record the event together with its time of onset.

### Quality control

To minimize errors, anthropometric measurements, clinical assessment and sample collection were done by the principal investigator or the trained research assistant. Weights were taken using the same weighing scale and height with the same height board for all study participants. To ensure adherence to the standard treatment protocols, checklists of the treatment protocols were pinned in the admission area to ensure the protocols are followed both at admission and during subsequent ward rounds, in as much as it was not part of the research to enforce compliance. Intern doctors who were at times the first health workers to see these patients were trained by the principal investigator on how to use the protocols. Laboratory results deemed important to the child’s management were availed to the attending doctors for definitive treatment of the study participant.

### Ethics approval and consent to participate

Ethics approvals were obtained from both the Faculty Research & Ethical Committee (FREC) and institutional Research Ethics Committee (REC) of Mbarara University of Science and Technology as IRB No. 06/04-15. Written informed consent was obtained from parents or guardians of all the study participants since all the study participants were less than 16 years old.

## Data analysis

### Study variables

The dependent variable was death while in hospital. This was presented as a binary variable with 0 = alive at the end of follow-up in hospital, and 1 = death in hospital at anytime during the study.

The independent variables included medical characteristics of children at enrolment, e.g. hypoxia, deep acidotic breathing, bradycardia, capillary refill > 2 seconds, temperature gradient in the limbs, weak pulse volume, reduced skin turgor, sunken eyes, decreased level of consciousness, hyponatremia, hypokalemia, hypoglycemia, HIV status of the patient, length of illness before admission, previous frequent episodes of diarrhea, evidence of bacteremia, oral candidiasis, edema, and pallor. Other independent variables were: nutritional, clinical, social, cultural and environmental determinants of severe malnutrition which as Nhamposa, etal [19] reported that if not addressed, may silently contribute to mortality in spite of these children receiving the best treatment. These included maternal illiteracy, paternal illiteracy, monthly family income of less than 50 USD, large family size with the number of children greater than 3 and inappropriate infant and young child feeding practices. Other factors that were studied as independent variables included, the length of time the child had been with his/her parents, the child’s immunization status and the time spent from onset of illness to arrival at the health facility. The socio-demographic characteristics like age of the patient, age of the parent/guardian, occupation of the parent/guardian, distance from health facility, ethnicity, and religion of the parent / caretaker were also considered as independent variables.

### Statistical methods

Data were entered in a Microsoft Excel 2007 database and all statistical analysis was performed using Stata® software (v. 11, College Station, Texas, USA). Relevant summary statistics were used to describe baseline characteristics of U5s, with respect to socio-demographic and medical factors, clinical presentation and examination findings, and laboratory parameters.

The primary outcome of the study was in-hospital mortality both within the first 48 hours and within the first 30 days of hospital admission. The proportion of children with severe acute malnutrition that died either in the first 48 hours of admission or within 30 days of in hospital study follow-up was expressed as a fraction of all the children included in the study.

Bivariate analysis, using chi squared test or Fischer’s exact test and poisson regression was performed to establish the association between independent variables with in-hospital mortality. Unadjusted incidence-rate-ratios (IRR) with their corresponding 95% confidence intervals (CI) were reported. A significance level of 5% was maintained. Variables with p-value <0∙1 were considered in multivariate analysis. Multivariate analysis models were fitted using poisson regression. A manual back-ward stepwise selection method was in model building. Adjusted incidence-rate-ratios with their corresponding 95% confidence intervals (CI) were reported for each variable in the final predictive multivariate model.

## Results

### Participants’ baseline characteristics

We enrolled a total of 122 children with SAM between June 2015 and August 2015.

Of these, 71 (58.2%) were males with a median age [IQR] of 15 months [11–24]. A total of 91 (74.6%) children were less than 24 months. A total of 87 (71.3%) participants had received medical care from a peripheral unit before coming to the Regional Referral Hospital. (See Table 1)

**Table 1:** Baseline characteristics of participants.

Characteristic	n (%)*
Median age in months [IQR]	15 [11–24]
Age categories in months, n (%)	
<12	31 (25.4)
12–23	60 (49.2)
24–59	31 (25.4)
Gender	
Male	71 (58.2)
Female	51 (41.8)
District of residence,	
Mbarara	56 (45.9)
Bushenyi	18 (14.8)
Isingiro	26 (21.3)
Kiruhura/Lyantonde/Rakai/Sembabule	16 (13.1)
Other	6 (4.9)
Relationship with informant	
Mother	101 (82.8)
Grandparent	15 (12.3)
Guardian	6 (4.9)
Pre-hospital care from a peripheral unit	87 (71.3)
Formal referral to hospital (N = 87)	45 (51.7)

Total observations, **N = 122,** unless stated otherwise

**Fig 1:**
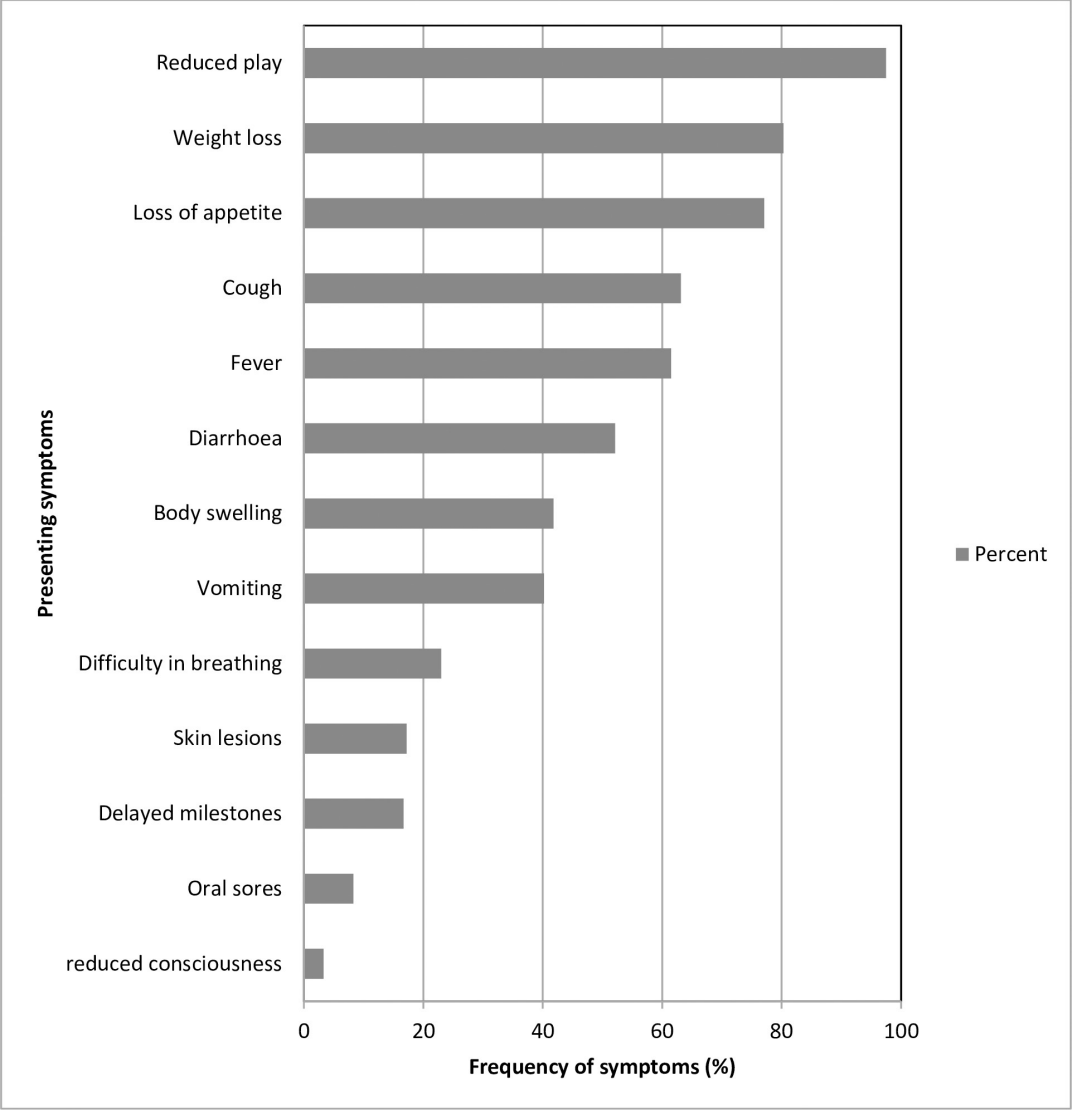
Presenting symptoms of the participants at admission.

The most common symptom at admission was reduced play (97.5%) followed by weight loss (80.3%) and the least frequent symptom was reduced consciousness (3.3%). (See Fig 1)

**Fig 2:**
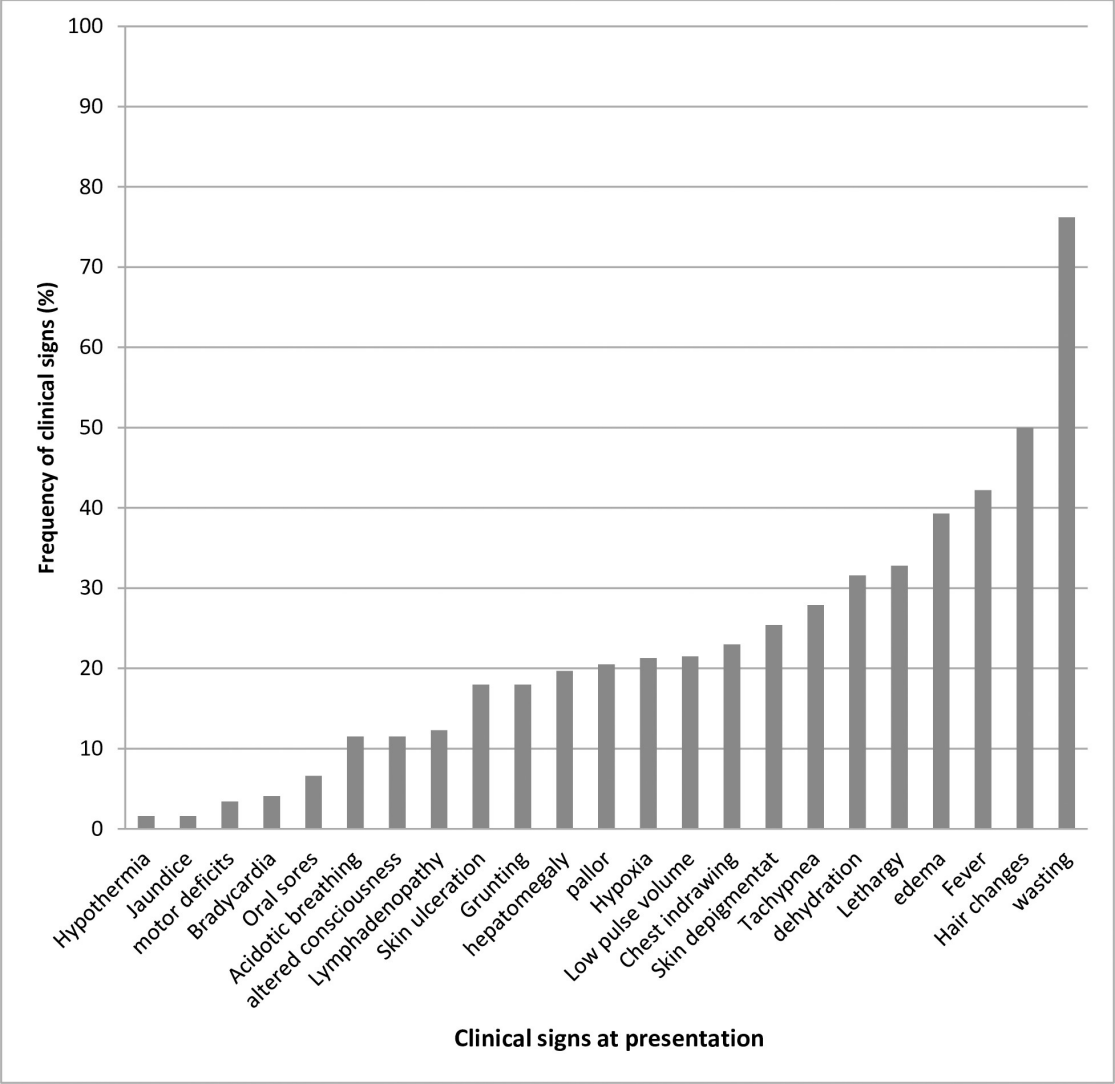
Presenting clinical signs of the partcipants at admission.

On clinical examination, most of the participants enrolled were wasted at presentation (76.2%) while only a small percentage had hypothermia (1.6%). All participants had features of SAM at presentation. Of the 122 participants, 48 (39.3%) had bilateral edema at presentation Fig 2. 97 (79.5%) had comorbidities with 54 (44.3%) of which having one comorbidity while 43 (35.2%) had more than one comorbidity. The commonest comorbidity at admission was Diarrheal illness (41.2%) while the least comorbidity was HIV (7.4%). Other comorbidities at admission included, Pneumonia (26.8%), Malaria (26.8%), severe Anemia (9.3%), severe dehydration (12.7%), and septic shock (11.3%). (See Fig 2)

A total of 25 (20.5%) participants had a positive malaria test, and only 9 (7.4%) participants had a positive HIV rapid test. Eight (6.6%) participants had hypoglycemia, 72 (59%) had anemia and 38 (31.1%) had a positive blood culture. (See Table 2)

**Table 2:** Laboratory parameters of participants at baseline.

Laboratory test.	n (%)
Serum Sodium	
Normal	77 (63.1)
High(> 145 mmol/l)	5 (4.1)
Low (< 135 mmol/l)	40 (32.8)
Serum Potassium	
Normal	91 (74.6)
High(> 5.5 mmol/l)	16 (13.1)
Low (< 3.5 mmol/l)	15 (12.3)
Blood culture	
Growth	38 (32.0)
No growth	84 (68.0)
Staph aureus	31 (81.6)
Strep pyogenes	1 (2.6)
E.Coli	3 (7.9)
Klebsiella	3 (7.9)
Malaria test	
Positive	25 (20.5)
Negative	97 (79.5)
HIV test	
Positive	9 (7.4)
Negative	113 (92.6)
Blood glucose	
Normal	112 (91.8)
High (> 8mmol/l)	2 (1.6)
Low (> 3mmol/l)	8 (6.6)
White cell count	
Normal	85 (69.7)
High (> 12x10^9^)	33 (27.1)
Low (< 4x10^9^)	4 (3.3)
Hemoglobin	
Normal(> 11.5g/dl)	50 (41.0)
Low (< 11.5g/dl)	72 (59.0)

### Mortality in hospital

Of the 122 participants that were successfully enrolled and followed up, 13 (10.7%; 95% CI: 5.1–16.2) of them died while in hospital, and 7 (5.7% [95% CI 1.6–9.9]) of the children who died in hospital, died in the first 48 hours.

### Predictors of in-hospital mortality

At bivariate analysis, under-fives with hypothermia had an 11-fold unadjusted risk of mortality when compared to under-fives who did not have hypothermia. Under-fives who had received intravenous fluid had twice unadjusted risk of mortality when compared to those who had not received intravenous fluid. (See Table 3) Other factors which were significantly associated with mortality on bivariate analysis are shown in Table 3.

**Table 3:** Results of bivariate analysis for predictors of in-hospital child mortality in SAM.

Characteristic (N = 122	Alive, n (%)	Dead,	Unadjusted IRR[95%CI]	P value
		n (%)		
Age in months				
<12	30 (96.8)	1 (3.2)	1	
12–23	50 (83.3)	10 (16.7)	5.2 [0.7–40.3]	0.117
24–59	29 (93.6)	2 (6.5)	2.0 [0.2–22.1]	0.571
MUAC				
<11.5cm	63 (84.0)	12 (16.0)	7.5 [0.98–57.84]	0.053
>11.5cm	46 (97.9)	1 (2.1)	1	
Hypothermia				
Yes	0 (0)	2 (100.0)	10.9 [2.42–49.23]	**0.002**
No	109 (90.8)	11 (9.2)	1	
Oral sores				
Yes	5 (62.5)	3 (37.5)	4.3 [1.18–15.53]	**0.027**
No	104 (91.2)	10 (8.6)	1	
Bradycardia				
Yes	2 (40.0)	3 (60.0)	7.0 [1.93–25.51]	**0.003**
No	107 (91.5)	10 (8.6)	1	
Pulse volume				
Weak	19 (73.1)	7 (26.9)	5.12 [1.62–16.12]	**0.005**
Normal	91 (94.8)	5 (5.3)	1	
Temp gradient				
Yes	5 (45.5)	6 (54.6)	8.6 [2.91–25.74]	**0.000**
No	104 (93.7)	7 (6.3)	1	
Altered consciousness				
Yes	10 (93.5)	7 (6.5)	6.6 [2.22–19.67]	**0.001**
No	8 (57.1)	6 (42.9)	1	
HIV				
Yes	7 (77.8)	2 (22.2)	2.6 [0.57–11.69]	0.216
No	102 (90.3)	11 (9.7)	1	
septic shock				
Yes	5 (45.4)	6 (54.6)	2.8 [2.91–25.74]	**0.000**
No	104 (93.7)	7 (6.3)	1	
comorbidities				
Yes	86 (88.7)	11 (11.3)	1.4 [0.31–6.40]	0.650
No	23 (92.0)	2 (8.0)	1	
Hypoglycemia				
Yes	7 (70)	3 (30.0)	3.4 [0.92–12.21]	**0.066**
No	102 (91.1)	10 (8.9)	1	
Bacteremia				
Yes	33 (86.8)	5 (13.2)	1.4 [0.45–4.22]	0.571
No	76 (90.5)	8 (9.5)	1	
Transfused				
Yes	6 (75.0)	2 (25.0)	1.0 [0.48–2.56]	0.180
No	104 (91.2)	10 (8.9)	1	
Administered IV fluids				
Yes	14 (63.6)	8 (36.4)	2.2 [1.00–3.40]	**0.000**
No	95 (96.0)	4 (4.0)	1	

In multivariate analysis, only the administration of IV fluids was significantly associated with mortality. Under-fives who had received intravenous fluid had seven times adjusted risk of mortality when compared to those who had not received intravenous fluids. There was a tendency towards increased risk of mortality for children who had a MUAC of less than 11.5cm and oral sores at examination although it did not reach statistical significance. (See Table 4)

**Table 4:** Results of Multivariate analysis for predictors of in-hospital child mortality in SAM.

Characteristic (N = 122)	Alive, n (%)	Dead, n (%)	Adjusted	P value
			IRR [95%CI]	
MUAC				
<11.5cm	63 (84.0)	12 (16.0)	6.1 [0.77–48.08]	0.087
≥11.5cm	46 (97.9)	1 (2.1)	1	
Received IV fluids				
Yes	14 (63.6)	8 (36.4)	7.2 [2.14–24.08]	**0.001**
No	95 (96.0)	4 (4.0)	1	
Oral sores				
Yes	5 (62.5)	3 (37.5)	3.2 [0.70–15.07]	0.134
No	104 (91.2)	10 (8.8)	1	

## Discussion

The proportion of children admitted with SAM who died while in hospital was 10.7%. Those who died in the first 48 hours of admission made up 5.7%. This overall in hospital mortality is lower than what has been reported in previous studies done in Ugandan settings [10, 11, 20]. This was probably because our study enrolled fewer participants compared to what Bachou et al (2006) at Mulago Hospital, Nyeko et al (2016) at Lacor Hospital and Maitland et al (2006) at Kilifi Hospital enrolled in their respective studies [10, 11, 20]. Nevertheless, our results are consistent with the already reported failure of the WHO treatment guidelines in improving outcomes in children with SAM [13]. This failure has been previously attributed to HIV infection, lack of maternal participation in the feeding program, inadequate care and prescription errors, and over prescription of intravenous therapies and blood transfusion [9, 11, 12, 14–16]. Another study also attributed the high mortality in children admitted with SAM to the WHO identified danger signs; lethargy, hypoglycemia and hypothermia as well as bradycardia, capillary refill greater than 2 seconds, weak pulse volume and impaired level of consciousness [10].

We found the administration of IV fluids to be an independent predictor of in-hospital mortality in children 6 months to 5 years with SAM at MRRH. This association has been reported before, with infusion of IV fluids being associated with excess deaths among children hospitalized with severe malnutrition and with these deaths being attributed to possible fluid overload [11, 21]. In our study, a total of 8 out of the 22 participants who received IV fluids died representing a case fatality of 36.4%. All these participants died within three days of receiving IV fluids further affirming a possible contribution of fluid overload to this mortality.

An alternative explanation could be the possibility that IV fluids were administered when it was already too late for the children to survive. According to WHO malnutrition guidelines that our study followed, fluid resuscitation should be given only if all of the following parameters are present; a weak, fast pulse, cold peripheries, a capillary refilling time (CRT) of > 3 seconds plus signs of impaired consciousness [22]. These features would be considered by pediatric life-support providers as constituting a very advanced state of shock, when outcome is generally poor [23]. Indeed a study done in South Africa noted that the WHO guidelines, though relatively simple to implement do not make much difference among severe acute malnutrition cases admitted in critical condition [24]. In our study, most of the patients came to hospital having been ill for more than two weeks with almost half of them having been referred from peripheral health facilities. A total of 40% of the children who received IV fluids during the study had shock at admission. It is possible that these children could have been in shock throughout the duration of their transportation to hospital. Indeed, a study on early goal-directed therapy in treatment of severe sepsis and septic shock demonstrated that for every hour that shock is left uncorrected, there is a doubling of mortality [25] Another big possibility is that the children admitted with SAM with edema could have had hypoalbuminemia. We did not set out to determine the serum albumin levels of these children because of the previously long held view that there was not a causal link between the oedema of malnutrition and the low plasma oncotic pressure induced by hypoalbuminaemia [26].However, a recent Reanalysis of this evidence and a review of the literature has shown that this evidence which, influenced the recommendations for treating children with severe acute malnutrition [22], was a mistaken conclusion and that the oedema is linked to hypoalbuminaemia [27] Malcolm (2015) further noted in his study that children with severe albumin deficiency from any cause continuously ‘struggle’ physiologically to maintain their blood volume by driving hormonal pathways that are normally only called upon in a crisis. They have no mechanisms in reserve; the mildest extra stress can rapidly precipitate severe shock. [27] It is possible that this was happening in the children in our study by the time they were admitted to hospital. Malcolm (2015) further reported that, if such children were treated with just 30 ml/kg over 2 hours of half-strength Darrow's solution with 5% dextrose (hypotonic crystalloid; sodium 61 mmol/l), as recommended by the WHO guidelines they may show a transient improvement as the fluid was delivered, but they would then deteriorate as the water leaked away into the tissues, and would have a high chance of dying [27–31]

Another plausible reason for the high mortality among children who received IV fluids is that there could have been inadequate correction of shock by the type of IV fluids that were prescribed to the children during the study period. This would agree with a study that compared use of half strength Darrow’s solution with use of isotonic solution in children with SAM that reported inadequate correction of shock in all study arms, resulting in a decision to prematurely terminate the trial [23]. On the other hand, Alam *et al* (2009) in Bangladesh reported that children were able to tolerate up to 100 ml/kg of isotonic fluid given for 6 hours [32]. It’s important to note though, that this study dealt with cholera patients that had significant losses of fluid and not children with SAM. The above findings though would agree with what Raman etal (2014) reported that Marasmic children, whose hypovolaemic shock is caused by an acute loss of salt and water uncomplicated by hypoalbuminaemia, require an intravenous infusion of sufficient isotonic fluid to promptly restore the circulating blood volume [33]. This allows oxygen delivery and perfusion of the organs [33]. They further reported that a rapid 20-ml/kg bolus of an isotonic fluid with glucose, repeated as necessary, would fulfil these logical and physiologically-based criteria [33] this is not what the WHO guidelines recommend. It is also noteworthy that WHO makes no distinction between managing shock in SAM without edema and SAM with edema [27], despite the fact that mortality is linked directly to the degree of oedema [28, 29] and hypoalbuminemia [30, 31]

The WHO recognized danger signs of lethargy, hypothermia and hypoglycemia as well as other clinical signs that pointed to shock, like weak pulse volume, temperature gradient and altered consciousness were not independently associated with mortality as has been reported in other studies [10, 20, 24] nor was age less than twenty four months [35]. This could be because the proportions of patients with these features in the above studies [10, 34, 35] were significantly greater than in our study causing them to register more outcomes than we did. The few numbers of patients in our study with these features (weak pulse volume, temperature gradient and altered consciousness) reduced its power to determine their association with mortality. What is clear though is that although each of them was not able to independently predict mortality, they in combination all point to a septic shock like syndrome that requires IV fluids as part of the management which could explain why administration of IV fluids was independently associated with mortality. We concur with Maitland etal (2009) in Kenya that more research is needed on early identification and better supportive care of sepsis as well as evidence based fluid management strategies [36].

We did not find MUAC to be significantly associated with death as another study found [37] though it predicted death in our children at bivariate analysis. It is possible that it could have been a significant predictor of death had our study had an equally long period like the above study [37] that retrospectively studied records of children with SAM spanning over 8 years.

We did not find HIV infection to be significantly associated with mortality as has been reported other studies [20, 38]. This could be attributed to the fact that half of the children enrolled in the study in South Africa and 28% of the children enrolled in the study at lacor Hospital were HIV positive compared to only 7.4% in our study.

We did not find children with Hyponatremia, Hypokalemia, Hypoglycemia and Bacteremia at admission to be at risk for mortality during admission in hospital as was reported by another study [10]. This could be attributed to the fact that our study had low number of participants and took a short period compared to this study [10] which enrolled almost 8 times the number of participants in our study over a period of two years.

Our study had a number of strengths and limitations. Our study had several methodological advantages. Because it was a cohort study, we were able to assess the temporal relationship between predictors and outcomes. Selection bias was also eliminated because participants were obtained at hospital and selected on the basis of being admitted within a given time period. The nature of the outcome made it impossible that any patient had the outcome at the beginning of the study. The weakness however was that the three months period for the study meant that we had few numbers of children for our study which significantly affected its power. Also, the fact we did not set out from the outset to study levels of creatinine in our study as well as Metabolic acidosis that were found in a study in a similar setting to be associated with late mortality in the under five children [10] could have affected our outcomes. Also the fact that we did not study serum albumin levels in these children yet edema in these children has now been found to cause hypoalbuminemia [27] which is linked to mortality [30, 31] could have affected our outcomes.

## Conclusion

The proportion of under five children with SAM who die during admission at Mbarara Regional Referral Hospital is high. Administration of intravenous fluids is a risk factor for mortality, regardless of other clinical and socio demographic factors. There is need to study hypoalbuminemia as a possible cause of severe shock in children with SAM with edema. A randomized controlled clinical trial needs to be conducted in our setting to compare the use of isotonic fluid and hypotonic fluids in management of severely malnourished children with shock. While Administration of intravenous fluids is still the main stay of managing severely malnourished children with shock, there is urgent need to review the present guidance on use of fluids in these children with special emphasis on the parameters presently used to assess children for shock as well as other possible causes of shock. This will help in diagnosing and managing shock in these children when it is still early.

## Supporting information

S1 Dataset Data set for Predictors of In-hospital mortality in children with SAM manuscript. (XLS)

## List of abbreviations

AIDS: Acquired Immune Deficiency Syndrome
AIRR: Adjusted Incidence Risk Ratio
HIV: Human Immuno deficiency virus
MUAC: Mid upper arm circumference
MUST: Mbarara University of Science and Technology
MRRH: Mbarara Regional Referral Hospital
NGT: Naso gastric tube
Resomal: Rehydration solution for malnutrition
RL: Ringer’s Lactate
SD: Standard deviation
UDHS: Uganda Demographic Health Survey
UTI: Urinary tract infection
WBC: white blood cell count
WHO: World Health Organization
WHZ: Weight for Height Z score
UNICEF: United Nations Children’s Fund

## References

[1] Mehta NM, Corkins MR, Lyman B, Malone A, Goday PS, Carney LN, et al Defining Pediatric Malnutrition A Paradigm Shift Toward Etiology-Related Definitions. Journal of Parenteral and Enteral Nutrition. 2013:0148607113479972. 10.1177/0148607113479972 23528324

[2] UNICEF. The state of the world's children 2008: Child survival: Unicef; 2007.

[3] Leroy JL, Ruel MT, Habicht J-P, Frongillo EA. Linear growth deficit continues to accumulate beyond the first 1000 days in low-and middle-income countries. 2014. 10.3945/jn.114.191981 24944283

[4] Organization WH. World health statistics 2010: World Health Organization; 2010.

[5] Saaka M, Osman SM, Amponsem A, Ziem JB, Abdul-Mumin A, Akanbong P, et al Treatment Outcome of Severe Acute Malnutrition Cases at the Tamale Teaching Hospital. Journal of Nutrition and Metabolism. 2015;2015. 10.1155/2015/641784 PMC4433717 26064678

[6] UNICEF. WHO, World Bank Levels and trends in child malnutrition Joint child malnutrition estimates New York, NY: United Nations International Children’s Fund 2014.

[7] UDHS I. Uganda demographic and health survey. Uganda Bureau of Statistics, Kampala Uganda 2011.

[8] Ashworth A, Ashworth A, Khanum S, Schofield C. Guidelines for the inpatient treatment of severely malnourished children: World Health Organization; 2003.

[9] Deen JL, Funk M, Guevara VC, Saloojee H, Doe JY, Palmer A, et al Implementation of WHO guidelines on management of severe malnutrition in hospitals in Africa. Bulletin of the World Health Organization. 2003;81:237–45. 12764489 PMC2572430

[10] Maitland K, Berkley JA, Shebbe M, Peshu N, English M, Newton CRC. Children with severe malnutrition: can those at highest risk of death be identified with the WHO protocol? PLoS medicine. 2006;3(12):e500 10.1371/journal.pmed.0030500 17194194 PMC1716191

[11] Bachou H, Tumwine JK, Mwadime RK, Tylleskär T. Risk factors in hospital deaths in severely malnourished children in Kampala, Uganda. BMC pediatrics. 2006;6(1):7. 16542415 10.1186/1471-2431-6-7 PMC1472687

[12] Fergusson P, Tomkins A. HIV prevalence and mortality among children undergoing treatment for severe acute malnutrition in sub-Saharan Africa: a systematic review and meta-analysis. Transactions of the Royal Society of Tropical Medicine and Hygiene. 2009;103(6):541–8. 10.1016/j.trstmh.2008.10.029 19058824

[13] Kerac M, Bunn J, Chagaluka G, Bahwere P, Tomkins A, Collins S, et al Follow-up of post-discharge growth and mortality after treatment for severe acute malnutrition (FuSAM study): a prospective cohort study. PloS one. 2014;9(6):e96030 10.1371/journal.pone.0096030 24892281 PMC4043484

[14] Nathoo K, Bannerman C, Pirie D. Pattern of admissions to the paediatric medical wards (1995 to 1996) at Harare Hospital, Zimbabwe. The Central African journal of medicine. 1999;45(10):258–63. 10.4314/cajm.v45i10.8496 10823229

[15] Karaolis N, Jackson D, Ashworth A, Sanders D, Sogaula N, McCoy D, et al WHO guidelines for severe malnutrition: are they feasible in rural African hospitals? Archives of disease in childhood. 2007;92(3):198–204. 10.1136/adc.2005.087346 16670119 PMC2083437

[16] Puoane T, Sanders D, Ashworth A, Chopra M, Strasser S, Mccoy D. Improving the hospital management of malnourished children by participatory research. International journal for quality in health care. 2004;16(1):31–40. 10.1093/intqhc/mzh002 15020558

[17] Adebisi YA, Ibrahim K, Lucero-Prisno DE III, Ekpenyong A, Micheal AI, Chinemelum IG, et al Prevalence and Socio-economic Impacts of Malnutrition Among Children in Uganda. Nutrition and Metabolic Insights. 2019;12:1178638819887398. 10.1177/1178638819887398 PMC6878600 31802887

[18] WHO. Management of severe malnutrition: a manual for physicians and other senior health workers1999.

[19] Nhampossa T, Sigaúque B, Machevo S, Macete E, Alonso P, Bassat Q, et al Severe malnutrition among children under the age of 5 years admitted to a rural district hospital in southern Mozambique. Public health nutrition. 2013;16(09):1565–74. 23635423 10.1017/S1368980013001080 PMC10271629

[20] Nyeko R, Calbi V, Ssegujja BO, Ayot GF. Treatment outcome among children under-five years hospitalized with severe acute malnutrition in St. Mary’s hospital Lacor, Northern Uganda. BMC Nutrition. 2016;2(1):19.

[21] Gebremichael M, Bezabih AM, Tsadik M. Treatment outcomes and associated risk factors of severely malnourished under five children admitted to therapeutic feeding centers of Mekelle City, Northern Ethiopia. Open Access Library Journal. 2014;1(04):1.

[22] Ashworth A, Chopra M, McCoy D, Sanders D, Jackson D, Karaolis N, et al WHO guidelines for management of severe malnutrition in rural South African hospitals: effect on case fatality and the influence of operational factors. The Lancet. 2004;363(9415):1110–5. 10.1016/S0140-6736(04)15894-7 15064029

[23] Akech SO, Karisa J, Nakamya P, Boga M, Maitland K. Phase II trial of isotonic fluid resuscitation in Kenyan children with severe malnutrition and hypovolaemia. BMC pediatrics. 2010;10(1):71. 20923577 10.1186/1471-2431-10-71 PMC2973932

[24] Muzigaba M, Van Wyk B, Puoane T. Management of severe acute malnutrition in children under 5 years through the lens of health care workers in two rural South African hospitals. African journal of primary health care & family medicine. 2018;10(1):1–8. 10.4102/phcfm.v10i1.1547 PMC5803520 29415550

[25] Rivers E, Nguyen B, Havstad S, Ressler J, Muzzin A, Knoblich B, et al Early goal-directed therapy in the treatment of severe sepsis and septic shock. New England Journal of Medicine. 2001;345(19):1368–77. 10.1056/NEJMoa010307 11794169

[26] Golden MN, Golden B, Jackson A. Albumin and nutritional oedema. The Lancet. 1980;315(8160):114–6. 10.1016/s0140-6736(80)90603-0 6101456

[27] Coulthard M G. Oedema in kwashiorkor is caused by hypoalbuminaemia. Paediatrics and international child health. 2015;35(2):83–9. 10.1179/2046905514Y.0000000154 25223408 PMC4462841

[28] Whitehead R, Frood J, Poskitt E. Value of serum-albumin measurements in nutritional surveys: A reappraisal. The Lancet. 1971;298(7719):287–9. 10.1016/s0140-6736(71)91334-1 4104977

[29] Williams CD. A nutritional disease of childhood associated with a maize diet. Archives of disease in childhood. 1933;8(48):423 10.1136/adc.8.48.423 21031941 PMC1975318

[30] Brasseur D, Hennart P, Dramaix M, Bahwere P, Donnen P, Tonglet R, et al Biological risk factors for fatal protein energy malnutrition in hospitalized children in Zaire. Journal of pediatric gastroenterology and nutrition. 1994;18(2):220–4. 10.1097/00005176-199402000-00016 8014771

[31] Van Der Westhuysen J, Kanengoni E, Jones J, Van Niekerk C. Plasma renin activity in oedematous and marasmic children with protein energy malnutrition. South African Medical Journal = Suid-afrikaanse Tydskrif vir Geneeskunde. 1975;49(42):1729–31. 810900

[32] Alam NH, Islam S, Sattar S, Monira S, Desjeux J-F. Safety of rapid intravenous rehydration and comparative efficacy of 3 oral rehydration solutions in the treatment of severely malnourished children with dehydrating cholera. Journal of pediatric gastroenterology and nutrition. 2009;48(3):318–27. 10.1097/mpg.0b013e318180af27 19274788

[33] Raman S, Peters MJ. Fluid management in the critically ill child. Pediatric Nephrology. 2014;29(1):23–34. 10.1007/s00467-013-2412-0 23361311

[34] Talbert A, Thuo N, Karisa J, Chesaro C, Ohuma E, Ignas J, et al Diarrhoea complicating severe acute malnutrition in Kenyan children: a prospective descriptive study of risk factors and outcome. PloS one. 2012;7(6):e38321 10.1371/journal.pone.0038321 22675542 PMC3366921

[35] Jarso H, Workicho A, Alemseged F. Survival status and predictors of mortality in severely malnourished children admitted to Jimma University Specialized Hospital from 2010 to 2012, Jimma, Ethiopia: a retrospective longitudinal study. BMC pediatrics. 2015;15(1):76. 26174805 10.1186/s12887-015-0398-4 PMC4502938

[36] Maitland K. Severe malnutrition: therapeutic challenges and treatment of hypovolaemic shock. Proc Nutr Soc. 2009;68:274–80. 10.1017/S0029665109001359 19490738

[37] Chiabi A, Mbanga C, Mah E, Nguefack Dongmo F, Nguefack S, Fru F, et al Weight-for-height z score and mid-upper arm circumference as predictors of mortality in children with severe acute malnutrition. Journal of tropical pediatrics. 2016;63(4):260–6. 10.1093/tropej/fmw083 28082667

[38] De Maayer T, Saloojee H. Clinical outcomes of severe malnutrition in a high tuberculosis and HIV setting. Archives of disease in childhood. 2011;96(6):560–4. 10.1136/adc.2010.205039 21310895

